# Transcriptome analysis of metabolic pathways associated with oil accumulation in developing seed kernels of *Styrax tonkinensis*, a woody biodiesel species

**DOI:** 10.1186/s12870-020-2327-4

**Published:** 2020-03-18

**Authors:** Qikui Wu, Yuanyuan Cao, Chen Chen, Zhenzhou Gao, Fangyuan Yu, Robert D. Guy

**Affiliations:** 1 Collaborative Innovation Centre of Sustainable Forestry in Southern China, College of Forest Science, Nanjing Forestry University, 159 Longpan Road, Nanjing, 210037 Jiangsu China; 2 Department of Forest and Conservation Sciences, Faculty of Forestry, University of British Columbia, 2424 Main Mall, Vancouver, BC V6T 1Z4 Canada

**Keywords:** *Styrax tonkinensis*, Biodiesel, Oil accumulation, Metabolic pathways, Transcriptome analysis

## Abstract

**Background:**

*Styrax tonkinensis* (Pierre) Craib ex Hartwich has great potential as a woody biodiesel species having seed kernels with high oil content, excellent fatty acid composition and good fuel properties. However, no transcriptome information is available on the molecular regulatory mechanism of oil accumulation in developing *S. tonkinensis* kernels.

**Results:**

The dynamic patterns of oil content and fatty acid composition at 11 time points from 50 to 150 days after flowering (DAF) were analyzed. The percent oil content showed an up-down-up pattern, with yield and degree of unsaturation peaking on or after 140 DAF. Four time points (50, 70, 100, and 130 DAF) were selected for Illumina transcriptome sequencing. Approximately 73 million high quality clean reads were generated, and then assembled into 168,207 unigenes with a mean length of 854 bp. There were 5916 genes that were differentially expressed between different time points. These differentially expressed genes were grouped into 9 clusters based on their expression patterns. Expression patterns of a subset of 12 unigenes were confirmed by qRT-PCR. Based on their functional annotation through the Basic Local Alignment Search Tool and publicly available protein databases, specific unigenes encoding key enzymes, transmembrane transporters, and transcription factors associated with oil accumulation were determined. Three main patterns of expression were evident. Most unigenes peaked at 70 DAF, coincident with a rapid increase in oil content during kernel development. Unigenes with high expression at 50 DAF were associated with plastid formation and earlier stages of oil synthesis, including pyruvate and acetyl-CoA formation. Unigenes associated with triacylglycerol biosynthesis and oil body development peaked at 100 or 130 DAF.

**Conclusions:**

Transcriptome changes during oil accumulation show a distinct temporal trend with few abrupt transitions. Expression profiles suggest that acetyl-CoA formation for oil biosynthesis is both directly from pyruvate and indirectly via acetaldehyde, and indicate that the main carbon source for fatty acid biosynthesis is triosephosphate originating from phosphohexose outside the plastid. Different *sn-*glycerol-3-phosphate acyltransferases are implicated in diacylglycerol biosynthesis at early versus late stages of oil accumulation. Triacylglycerol biosynthesis may be accomplished by both diacylglycerol and by phospholipid:diacylglycerol acyltransferases.

## Background

Biodiesel, one of the most important sources of renewable and clean energy worldwide, was first successfully produced in 1853 from vegetable oil, the first-generation biodiesel feedstock [1, 2]. Second-generation biodiesel feedstock is derived from non-edible sources, such as oils from *Millettia pinnata* (L.) Panigrahi, *Jatropha curcas* L. and microalgae, solving the ‘food versus fuel’ dispute and decreasing production costs, especially the cost of raw materials [3]. Recently, attention has been given to woody plants with low cost and ease of cultivation as potential sources of biodiesel feedstock [4, 5].

*Styrax tonkinensis* (Pierre) Craib ex Hartwich, a member of the *Styracaceae*, is native to secondary rainforests in northern Laos and Vietnam, and introduced into West Africa and southern China [6]. This plant is an economically important tree species because of its timber, benzoin resin, seed oil and ornamental value [7–10]. In China, *S. tonkinensis* has great potential as a woody biodiesel species having seed kernels with high oil content (54.86%), excellent fatty acid composition and good fuel properties [9]. To date, related studies on *S. tonkinensis* have focused mostly on cultivation, wood production, benzoin resin production, and biofuel production [11–14]. To study development of *S. tonkinensis* kernels, Zhang et al. [15] collected samples from 30 to 140 days after flowering (DAF) for analyses of morphometric indicators (longitudinal sections, weight and water content), nutritional components (oil, soluble sugar, starch, free amino-acid and soluble protein) and related enzyme activities. The dynamic pattern of oil accumulation in developing *S. tonkinensis* seed kernels has also been analyzed [15–17]. However, little is known about the molecular regulatory mechanism of oil accumulation in this species, and no RNA-seq datasets of any sort are available for any member of the *Styracaceae*. Understanding the molecular basis of oil accumulation is imperative for the development and enhancement of *S. tonkinensis* kernels as biodiesel feedstock.

In oil seeds, de novo oil biosynthesis, including fatty acid (FA) and triacylglycerol (TAG) biosynthesis, requires acetyl-CoA, ATP and reducing power. Key catalytic enzymes include the pyruvate dehydrogenase complex (PDC), the acetyl-CoA carboxylase (ACC) complex, the fatty acid synthase (FAS) complex, and acyl-CoA:DAG acyltransferase (DGAT) [18–20]. Zhang et al. [15] have previously shown that PDC and ACC activities support FA biosynthesis in developing *S. tonkinensis* kernels, and DGAT supports TAG biosynthesis. Different parts of the process are localized in different compartments; for example, glycolysis in the cytosol and plastids, FA biosynthesis in plastids, and TAG biosynthesis in endoplasmic reticulum (ER) [20]. Thus, there are many transmembrane transporters needed to mediate the transfer of essential metabolites between compartments in developing oil seeds [21–24].

Understanding of the pathways involved in oil accumulation during *S. tonkinensis* kernel development requires the exploration of functional genes, transcription factors and transmembrane transporters. RNA-seq, one of the next-generation sequencing (NGS) technologies, is widely used for exploring functional genes, constructing expression profiles and transcriptional regulatory pathways, and investigating comparative and evolutionary genomics [25–27]. It provides a readily available, cheap, and comprehensive method to acquire large-scale genomic and transcriptomic data for non-model plant species and has been used to analyze the transcriptomic profiles and construct summary overviews of FA and TAG biosynthesis during oil accumulation in woody oil plants, such as *Elaeis guineensis* Jacq., *Prunus sibirica* L., *Pistacia chinensis* Bunge, *Symplocos paniculata* Miq., and *Lindera glauca* (Siebold & Zucc.) Blume [28–32]. However, functional gene expression and regulatory profiles of oil biosynthesis and metabolism in *S. tonkinensis* have not yet been analyzed.

In this study, we explored changes in gene expression, oil content and FA composition during seed kernel development in *S. tonkinensis* over an extended time course. The aims of the study were to: (1) confirm the dynamic pattern of oil content and FA composition during kernel development; (2) assess transcriptional profiles at four time points leading up to kernel maturity; (3) identify functional unigenes encoding vital enzymes related to FA and TAG biosynthesis; and (4) confirm the metabolic pathways associated with oil accumulation in developing kernels indicated by functional unigenes.

## Results

### Dynamic patterns of oil content and fatty acid composition

**Fig. 1:**
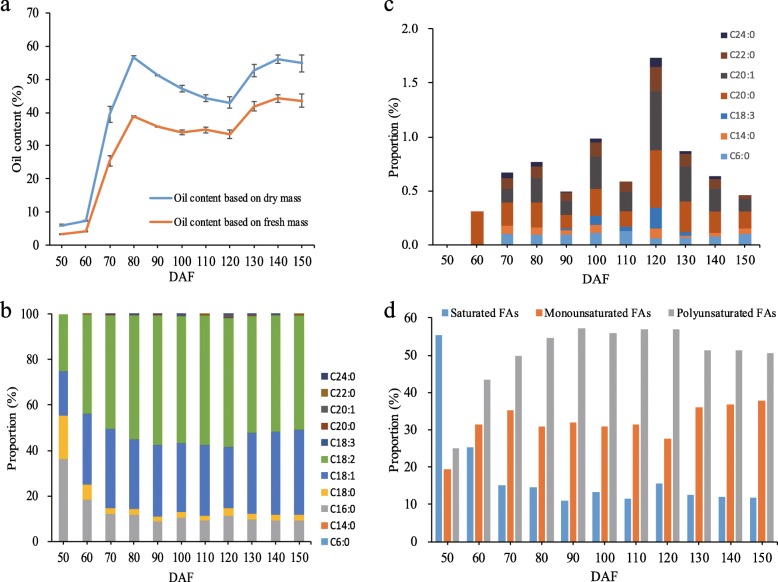
Changes in oil accumulation and fatty acid (FA) composition during *S. tonkinensis* kernel development. **a** Oil content based on dry mass and fresh mass of *S. tonkinensis* kernels relative to days after flowering (DAF). Data are means ± SD of three biological replicates. **b** Changes in FA composition. **c** Changes in minor FA composition during *S. tonkinensis* kernel development. **d** Relative proportions of saturated, monounsaturated and polyunsaturated FAs

In this study, the dynamic pattern of oil concentration based on fresh mass and dry mass during kernel development was evaluated (Fig. 1a). There was a dramatic increase in the per cent oil content from 60 to 70 days after flowering (DAF). Thereafter, per cent oil content followed an up-down-up pattern with peaks at 80 DAF and at kernel maturity. The final oil content represented about 44 and 56% of the kernel fresh mass and dry mass, respectively.

As shown in Fig. 1b, FA composition changed during kernel development. Among 11 detected FAs, there were four major FAs: C16:0 (palmitic acid), C18:0 (stearic acid), C18:1 (oleic acid) and C18:2 (linoleic acid). Their proportions at the earliest sampling time (50 DAF) were 36.2, 19.3, 19.5 and 25.1%, respectively. By 100 DAF, C16:0 and C18:0 had decreased to 10.2 and 2.5%, and C18:1 and C18:2 increased to 30.5 and 55.8%, respectively. Other minor FAs showed a different pattern, peaking overall at 120 DAF (Fig. 1c). Changes in the proportions of saturated, monounsaturated and polyunsaturated FAs (Fig. 1d) depended mainly on the four major FAs.

The biodiesel fuel properties, including density (ρ), kinematic viscosity (η), cetane number (CN), iodine value (IV), and cold filter plugging point (CFPP), were predicted based on the FA composition (Additional file 1). During *S. tonkinensis* kernel development, the estimated ρ ranged from 0.856 (90 DAF) to 0.868 (50 DAF) kg/m^3^ with an average of 0.859 kg/m^3^. The estimated η ranged from 3.35 (110 DAF) to 3.71 (50 DAF) mm^2^/s with an average of 3.42 mm^2^/s. Estimated CN ranged between 47.4 (90 DAF) and 56.7 (50 DAF) with an average of 49.1, and estimated IV was between 71.5 (50 DAF) and 122.8 (90 DAF) g/100 g with an average of 113.4 g/100 g. The estimated CFPP ranged from − 11.2 (90 DAF) to 8.49 (50 DAF) °C with an average of − 7.95 °C. The estimated average ρ, η, CN, IV, and CFPP values at kernel maturity (between 120 and 150 DAF) were 0.86 kg/m^3^, 3.40 mm^2^/s, 48.2, 118.3 g/100 g and − 9.80 °C, respectively.

### Sequence analysis and de novo transcriptome assembly

Based on the observed changes in oil content and quality reported in Fig. 1, and related changes in morphological and physiological indicators in previous studies [15, 17], we defined four stages of kernel development: an initial stage prior to the major rise in content (50–60 DAF), an active stage of rising oil content (60–80 DAF), a third stage with decreased oil content and high polyunsaturated FAs (80–120 DAF), and a final maturation stage with stable oil content and composition (120–150 DAF). To explore changes in transcription, we sampled these stages at 50, 70, 100 and 130 DAF. Three cDNA libraries were constructed from independent kernel RNA samples obtained at each of these four time points.

More than 5 GB raw data were respectively generated from every RNA-Seq sample. An average 72,584,106 (97.15%) of clean reads was obtained after removing the adaptor and low-quality reads (Table 1). A total of 365,312 contigs were obtained with a mean length of 1146 bp and GC content of 40.45% using Trinity software. Subsequently, a final contigs assembly produced 168,205 unigenes (N50: 1437) with a mean length of 854 bp. The number of unigenes assembled in this study was comparable to numbers assembled in studies of kernel development in other woody plants [29–32]. Of the unigenes we found, 42.25% (71,071) were 200–400 bp in length, 32.17% (54,119) were 400–1000 bp, 15.62% (26,268) were 1000–2000 bp, and 9.96% (6747) exceeded 2000 bp (Table 2).

**Table 1:** Statistics of raw data and clean data

Sample (DAF)	Raw data		Clean data			Clean ratio (reads) (%)
	Reads	Bases	Reads	Bases	Average length	
50	76,947,096	11,542,064,400	75,089,043	11,263,356,500	150	97.58
70	74,177,616	11,126,642,400	71,321,505	10,698,225,800	150	96.13
100	76,880,065	11,532,009,700	74,838,326	11,225,748,900	150	97.35
130	70,835,256	10,625,288,400	69,087,551	10,363,132,600	150	97.53

**Table 2:** Transcriptome assembly statistics

Items	Contigs	Unigenes
Total number	365,312	168,205
Total base (bp)	418,631,522	143,637,971
GC content (%)	40.45	40.55
Average length (bp)	1146	854
N50 (bp)	1952	1437
N90 (bp)	472	333
Range of length (bp)	201–16,802	201–16,802
200–400 bp	113,310	71,071
400–1000 bp	111,742	54,119
1000–2000 bp	76,754	26,268
≥2000 bp	63,506	16,747

### Functional annotation and classification

To annotate and identify putative functions, all the assembled unigenes were annotated using the Basic Local Alignment Search Tool (BLAST) program against five publicly available protein databases with a cut-off E-value of 10^− 5^. In total, there were 74,524 (44.30%), 63,788 (37.92%), 60,017 (35.81%), 49,305 (29.31%), and 20,424 (12.14%) unigenes that had significant hits with known proteins in non-redundant protein (NR), the Swiss-Prot protein (SwissProt), Gene Ontology (GO), Clusters of Orthologous Groups (COG), and the Kyoto Encyclopedia of Genes and Genomes (KEGG) databases, respectively. Of all the assembled unigenes, 89,387 (53.14%) had significant BLAST matches in at least one of the five databases, whereas 14,105 (8.39%) had significant hits in all databases. However, the remaining 78,818 (46.86%) unigenes had no significant annotation hit, either because they represent novel tissue-specific genes not in a database or because there were a large number of short sequences (71,071, 42.25%) lacking a characterized protein domain to get BLAST hits.

**Fig. 2:**
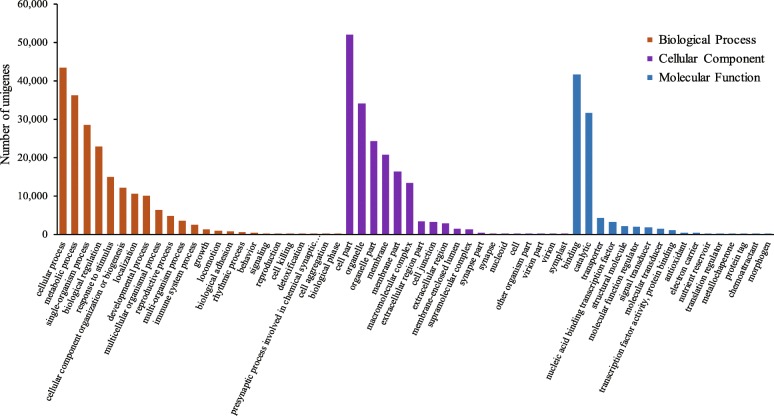
GO classifications of *S. tonkinensis* unigenes

A total of 60,017 assembled unigenes were assigned into three main GO functional categories (biological process, cellular component, and molecular function) and 60 sub-categories (Fig. 2). The biological process category was divided into 24 sub-categories. The two most abundant sub-categories were “cellular process” and “metabolic process”, which contained 43,503 unigenes (69.65%) and 36,285 unigenes (58.10%), respectively. The cellular component category was assigned into 19 sub-categories with the majority of unigenes in “cell part” (51,935, 83.16%), followed by “organelle” (34,131, 54.65%). The molecular function category was divided into 17 sub-categories, including the two most abundant sub-categories “binding” (41,690, 66.75%) and “catalytic” (31,649, 50.66%).

**Fig. 3:**
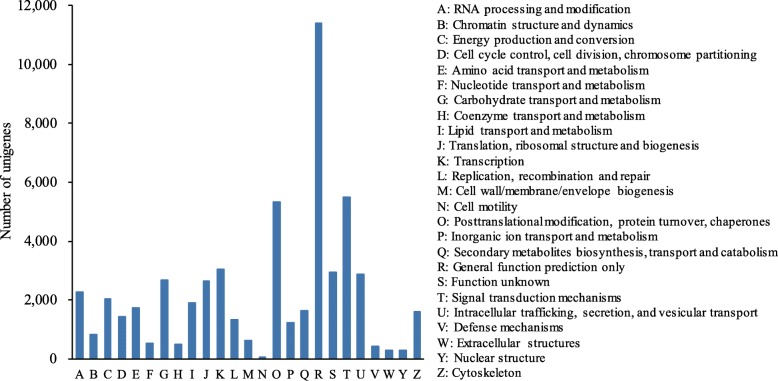
COG classifications of *S. tonkinensis* unigenes

A total of 49,305 unigenes were assigned into 25 COG classifications with the majority in “general function prediction only” (11,403, 23.13%), followed by “signal transduction mechanisms” (5485, 11.12%) and “posttranslational modification, protein turnover, chaperones” (5318, 10.79%) (Fig. 3). Furthermore, there were 1889 (3.83%) unigenes assigned into “lipid translations and metabolism”, suggesting candidates that may be related to oil accumulation in *S. tonkinensis* kernels.

**Fig. 4:**
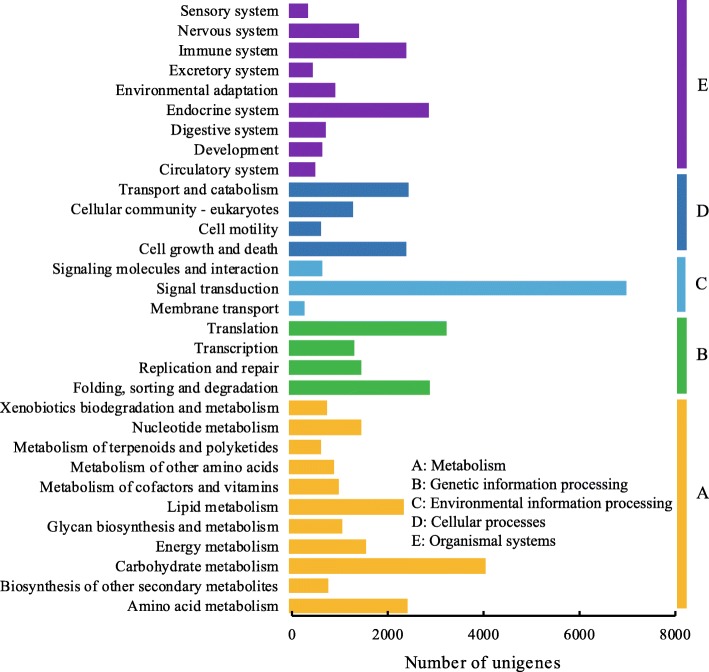
KEGG classifications and pathway assignment of *S. tonkinensis* unigenes

The KEGG classifications for the assembled unigenes were used to evaluate the completeness of the transcriptome library and the effectiveness of the annotation process [33], and a total of 20,424 unigenes were assigned into five KEGG categories, 31 sub-categories, and 427 KEGG pathways (Fig. 4). Of the 20,424 unigenes, approximately 2280 unigenes were mapped to 16 out of 17 lipid metabolic canonical pathways, the exception being “secondary bile acid biosynthesis”. Among them, “glycerophospholipid metabolism” had the highest number of unigenes (410), followed by “fatty acid degradation” (299 unigenes), “glycerolipid metabolism” (269 unigenes), and “fatty acid biosynthesis” (194 unigenes). In addition, other pathways related to lipid metabolism were “linoleic acid metabolism” (80 unigenes), “fatty acid elongation” (97 unigenes), “biosynthesis of unsaturated fatty acids” (122 unigenes), and “alpha-linolenic acid metabolism” (166 unigenes).

### Analysis of gene expression

**Fig. 5:**
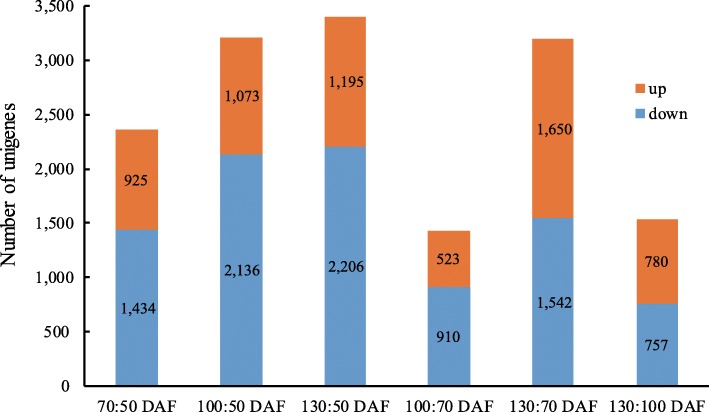
Number and distribution of up-regulated and down-regulated unigenes of *S. tonkinensis* seed kernels between different time points

To investigate the dynamic expression patterns of the specific genes associated with oil accumulation, the transcriptome profiles from different time points were compared. The Reads Per Kilobase Million Mapped Reads (RPKM) values were statistically analyzed to select different unigenes by using the DESeq method [34]. The up and down regulated unigenes between different time points were tallied (Fig. 5, Additional file 2). A total of 2359 unigenes had different expression patterns between 50 and 70 DAF with 925 up-regulated unigenes and 1434 down-regulated unigenes. A total of 3209 unigenes were expressed differentially at 100 DAF (50 DAF as control), with more down-regulated unigenes (2136) than up-regulated unigenes (1073). There were 3401 unigenes which had different expression patterns between 50 and 130 DAF, including 1195 up-regulated unigenes and 2206 down-regulated unigenes. A total of 1433 unigenes had different expression patterns between 70 and 100 DAF with 523 up-regulated unigenes and 910 down-regulated unigenes. A total of 3192 unigenes expressed differentially between 70 and 130 DAF with 1650 up-regulated unigenes and 1542 down-regulated unigenes. There were 1537 unigenes which had different expression patterns between 100 and 130 DAF, including 780 up-regulated unigenes and 757 down-regulated unigenes.

All 5916 differentially expressed genes (DEGs) were subject to a clustering analysis using Mfuzz to yield 9 clusters (Additional file 3). Cluster I, containing 1115 DEGs, consisted mainly of unigenes that had a rapid decrease in expression between 50 and 70 DAF, remaining low thereafter. The major pathway enrichment of unigenes in cluster I was focused on biosynthesis of amino acids, glycerolipid metabolism and starch and sucrose metabolism. Cluster II contained 1035 DEGs, representing the unigenes with continuous downward trends from 50 through to 100 DAF. The unigenes of cluster II were mainly enriched into the pathways of biosynthesis of amino acids, glycolysis/gluconeogenesis and purine metabolism. Cluster III, containing 839 DEGs, represented the unigenes with higher expression levels at both 50 and 70 DAF. The pathway enrichment of unigenes in cluster III was mainly focused on fatty acid biosynthesis, fatty acid metabolism, glycolysis/gluconeogenesis, the pentose phosphate pathway (PPP), protein processing in endoplasmic reticulum, pyruvate metabolism and the TCA cycle. Clusters IV and V, containing 485 and 580 DEGs respectively, both peaked at 70 DAF during the most active period of rising oil content. Cluster IV was enriched in unigenes for ABC transporters, amino sugar and nucleotide sugar metabolism, fatty acid biosynthesis and the PPP. Cluster V rose to the peak at 70 DAF more abruptly than cluster IV and was enriched in unigenes for glycerophospholipid metabolism, pyruvate metabolism and tyrosine metabolism. Cluster VI contained 226 DEGs, and represented the unigenes with expression levels that mostly continued to increase from 70 to 100 DAF, as oil accumulation was slowing down. This cluster was mainly enriched in the pathways of glycolysis/gluconeogenesis, pyruvate metabolism and ubiquitin mediated proteolysis. Cluster VII contained 339 DEGs, representing the unigenes that showed a more-or-less gradual increase during all of kernel development and peaking at either 100 or 130 DAF. Cluster VII was enriched in unigenes for alpha-linolenic acid metabolism, glycerolipid metabolism, glycerophospholipid metabolism, purine metabolism and ribosomal proteins. Cluster VIII contained 565 DEGs, representing the unigenes with somewhat up-down-up change trends during kernel development and was mainly enriched in the pathways of fatty acid metabolism, glycolysis/gluconeogenesis, protein processing in endoplasmic reticulum and starch and sucrose metabolism. Cluster IX, containing 731 unigenes, represented the unigenes with complex change trends and lower expression levels at 70 or 100 DAF. The pathway enrichment of unigenes in cluster IX was mainly focused on biosynthesis of amino acids, fatty acid degradation, fatty acid metabolism, glycerolipid metabolism, oxidative phosphorylation, phenylpropanoid biosynthesis, protein processing in endoplasmic reticulum and pyrimidine metabolism.

To validate the RNA-Seq results, relative expression and temporal transcription profiles of 12 identified key genes were analyzed by qRT-PCR. There was very good consistency between the relative expression levels of these genes and the RPKM comparative ratios (Additional file 4). Thus, the dynamic expression patterns of kernel unigenes uncovered by RNA-Seq can be considered reliable.

### Unigenes related to pyruvate and acetyl-CoA formation

Unigenes representing key enzymes in the glycolytic pathway and the pentose phosphate pathway (PPP) related to pyruvate and acetyl-CoA formation include ATP-dependent phosphofructokinase (PFK, EC: 2.7.1.11), pyrophosphate-dependent phosphofructokinase (PFP, EC: 2.7.1.90), pyruvate kinase (PK, EC: 2.7.1.40), acetyl-CoA synthetase (ACS, EC: 6.2.1.1), glucose-6-phosphate dehydrogenase (G6PD, EC: 1.1.1.49), 6-phosphogluconate dehydrogenase (PGD, EC: 1.1.1.44), and the PDC, including pyruvate dehydrogenase (PDC E1-α and PDC E1-β, EC: 1.2.4.1), dihydrolipoamide transacetylase (PDC E2, EC: 2.3.1.12), and dihydrolipoamide dehydrogenase (PDC E3, EC: 1.8.1.4).

**Fig. 6:**
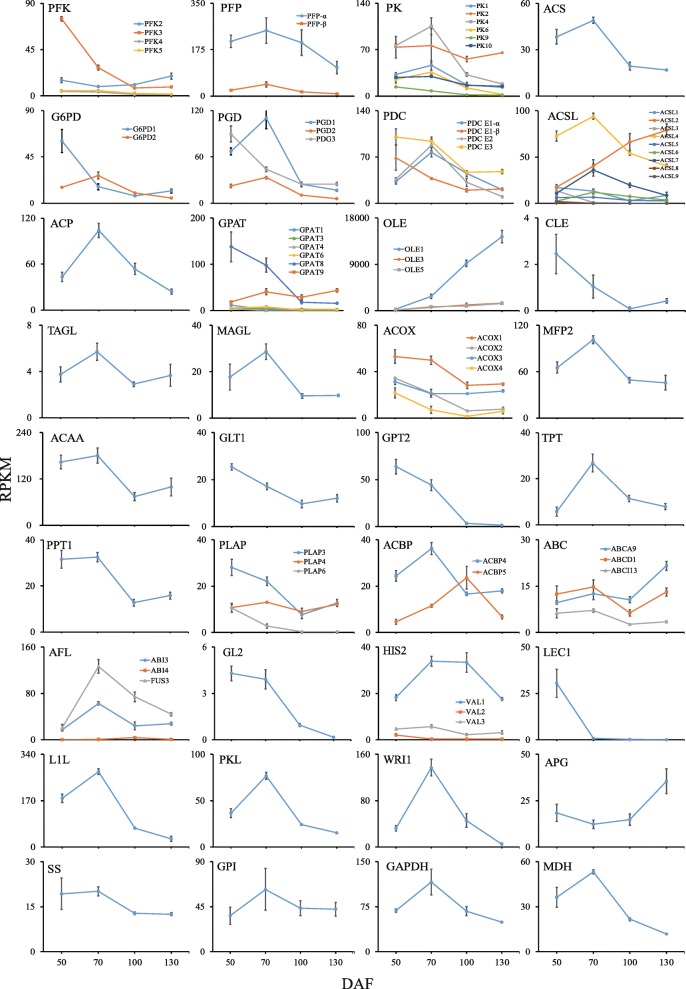
Dynamic patterns of related unigenes and transcription factors in the conversion of sucrose to triacylglycerol. Data are means ± SD of three biological replicates. Abbreviations: FPK, ATP-dependent phosphofructokinase; PFP, pyrophosphate-dependent phosphofructokinase; PK, pyruvate kinase; ACS, acetyl-CoA synthetase; G6PD, glucose-6-phosphate dehydrogenase; PGD, 6-phosphogluconate dehydrogenase; PDC, pyruvate dehydrogenase complex; ACSL, long-chain acyl-CoA synthetases; ACP, malonyl-acyl carrier protein; ACBP, acyl-CoA binding protein; GPAT, *sn*-G3P acyltransferase; OLE, oleosin; CLE, caleosin; ACOX, acyl-CoA oxidase; MF2, enoyl-CoA hydratase/3-hydroxyacyl-CoA dehydrogenase; ACAA, acetyl-CoA acyltransferase; TAGL, triacylglycerol lipase; MAGL, monoacylglycerol lipase; GLT1, glycolipid transporters; TPT, triose phosphate transporters; GPT2, glucose-6-phosphate transporters; PPT1, phosphoenolpyruvate transporters; PLAP, plastid-lipid-associated protein; ABC, ATP-binding cassette transporters; ABI3, abscisic acid insensitive 3; ABI4, abscisic acid insensitive 4; FUS3, fusca3; GL2, glabra 2; HSI2/VAL1, high-level expression of sugar-inducible gene 2; HSIL1/VAL2, HSI2-like1; HSIL1/VAL3, HSI2-like2; LEC1, leafy cotyledon 1; L1L, LEC1-like; PKL, pickle; WRI1, wrinkled 1; APG, glucose-1-phosphate adenylyltransferase; SS, starch synthase; GPI, glucose-6-phosphate isomerase; GAPDH, glyceraldehyde 3-phosphate dehydrogenase; MDH, malate dehydrogenase

Ten unigenes were identified to encode PFK, including two for PFK2, four for PFK3, two for PFK4, and two for PFK5. Nine unigenes were identified for PFP (six for PFP-α and three for PFP-β). Twenty-three unigenes were found to encode PK, which included nine for PK1, six for PK2, four for PK4, one for PK6, one for PK9, and two for PK10. Moreover, three unigenes were identified to encode ACS and 22 unigenes were found to encode four subunits of the PDC, including two for PDC E1-α, five for PDC E1-β, eight for PDC E2, and seven for PDC E3. Seven unigenes encoding G6PD (four for G6PD1 and three for G6PD2) and five unigenes encoding PGD (two for PGD1, one for PGD2, and two for PGD3) were identified. For each protein, the unigene with the highest RPKM value was selected to examine expression levels over time (Additional file 5). The selected unigenes showed two major expression patterns (Fig. 6), with peaking at 50 DAF (*pfk3*, *g6pd1*, *pgd3*, *pdc E1-β* and *pdc E3*), or at 70 DAF (*pfp*, *pk*, *acs*, *g6pd2*, *pdg1*, *pdg2*, *pdc E1-α* and *pdc E2*). All of these unigenes had a comparatively low expression level at the last two time points (100 and 130 DAF).

### Unigenes related to fatty acid biosynthesis

All unigenes of enzymes involved in FA biosynthesis were identified, including acetyl-CoA carboxylase (ACC, EC: 6.4.1.2; three subunits: ACC:carboxyltransferase (accA), biotin carboxyl carrier protein (accB), and biotin carboxylase (accC)), malonyl-CoA-ACP transacylase (MAT, EC: 2.3.1.39), and the FAS complex (3-oxoacyl-ACP synthase I (KASI, EC: 2.3.1.41), 3-ketoacyl-ACP synthase II (KASII, EC: 2.3.1.179), 3-ketoacyl-ACP synthase III (KASIII, EC: 2.3.1.180), 3-ketoacyl-ACP reductase (KAR, EC: 1.1.1.100), 3-hydroxyacyl-ACP dehydratase (HAD, EC: 4.2.1.59), and enoyl-ACP reductase (EAR, EC: 1.3.1.9)). In addition, the unigenes for enzymes involved in FA lengthening and desaturation were identified: stearoyl-ACP desaturase (SAD, EC: 1.14.19.2), acyl-ACP thioesterase (FAT, EC: 3.1.2.14), long-chain acyl-CoA synthetase (ACSL, EC: 6.2.1.3), lysophosphatidylcholine acyltransferase (LPCAT, EC: 2.3.1.23), fatty acid desaturase 2 (FAD2, EC: 1.14.19.6), and phospholipase A2 (PLA2, EC: 3.1.1.4).

Nine unigenes were found to encode ACC including three for accA, three for accB, and three for accC. One unigene was found for MAT. Thirty-eight unigenes were identified as essential cofactors of FAS components, including 20 for KAS (six for KASI, five for KASII, and nine for KASIII), eight for KAR, three for HAD, and seven for EAR. Six unigenes were found to encode SAD. Moreover, eight unigenes encoding FAT (two for FATA and six for FATB) and 21 unigenes encoding ACSL (three for ACSL1, three for ACSL2, one for ACSL3, seven for ACSL4, one for ACSL5, three for ACSL6, one for ACSL7, one for ACSL8, and one for ACSL9) were identified. Three unigenes were identified to encode FAD2, five for LPCAT1 and seven for PLA2. Additionally, eight unigenes for ACP were identified by the functional annotation analyses. The unigene expression trends of enzymes involved in FA biosynthesis were analyzed (Fig. 6). Except KAR and PLA2, all the FA biosynthesis unigenes had dynamic expression patterns similar to the unigenes related to pyruvate and acetyl-CoA formation, with peak expression at the second time point (70 DAF). The *fatb* unigene had low expression at the later stages (100 and 130 DAF), while *fad2* had low expression at the initial stage (50 DAF).

### Unigenes related to triacylglycerol and oil body biosynthesis

Five enzymes related to triacylglycerol (TAG) biosynthesis were identified: glycerol kinase (GK, EC: 2.7.1.30), *sn*-G3P acyltransferase (GPAT, EC: 2.3.1.15), lysophosphatidyl acyltransferase (LPAT, EC: 2.3.1.5), acyl-CoA:DAG acyltransferase (DGAT, EC: 2.3.1.20) and phospholipid:diacylglycerol acyltransferase (PDAT, EC: 2.3.1.158). We identified two unigenes for encoding GK, thirteen for GPAT (four for GPAT1, two for GPAT3, one for GPAT4, three for GPAT6, one for GPAT8, and two for GPAT9), eighteen for LPAT (five for LPAT1, five for LPAT2, three for LPAT4, and five for LPAT5), and seven for PAP. Nine unigenes for DGAT and seven for PDAT (six for PDAT1 and one for PDAT2) were identified. The change trends of unigenes related to TAG biosynthesis were complex (Additional file 5). GK had a downward trend during kernel development, while GPAT showed an up-down-up trend peaking at 70 DAF. LPAT and PAP had a similar change trend with a peak at 70 DAF, while PDAT and DGAT peaked at 100 and 130DAF, respectively.

Unigenes encoding the oil body membrane proteins oleosin (OLE), caleosin (CLE) and steroleosin (SLE) were analyzed. Nine unigenes encoding OLE were identified (six for OLE1, two for OLE3, and one for OLE5), but only one unigene encoding CLE was found, and none for SLE. With kernel development, *ole1* expression levels increased continuously but the other unigenes were low and did not show much change.

### Unigenes related to fatty acid and triacylglycerol degradation and metabolism

Unigenes related to FA and TAG degradation and metabolism included two TAG lipases (triacylglycerol lipase (TAGL, EC: 3.1.1.3) and monoacylglycerol lipase (MAGL, EC: 3.1.1.23), and three core enzymes of the β-oxidation pathway (acyl-CoA oxidase (ACOX, EC: 1.3.3.6), enoyl-CoA hydratase/3-hydroxyacyl-CoA dehydrogenase (MFP2, EC: 4.2.1.17, 1.1.1.35, 1.1.1.211), and acetyl-CoA acyltransferase (ACAA, EC: 2.3.1.16)). We identified four unigenes encoding TAGL, two encoding MAGL, nine encoding ACOX (one for ACOX1, four for ACOX2, three for ACOX3, and one for ACOX4), four encoding MFP2 and three encoding ACAA. The expression patterns of these unigenes peaked early in kernel development, at either 50 or 70 DAF (Fig. 6).

### Unigenes related to transmembrane transporters, transcription factors and carbon partitioning

We found unigenes for five transmembrane transporters of glycolytic metabolites and two for FA or acyl-CoA interchange between different organelles; namely, glycolipid transporters (GLT), glucose-6-phosphate transporters (GPT), triose phosphate transporters (TPT), phosphoenolpyruvate transporters (PPT), plastid-lipid-associated protein (PLAP), acyl-CoA binding protein (ACBP) and ATP-binding cassette transporters (ABC). In this study, five, nine, twenty-nine, five, and eleven unigenes were identified to encode GLT1, GPT2, TPT, PPT1, and PLAP (six for PLAP3, three for PLAP4, and two for PLAP6), respectively. Expression levels of *glt1*, *gpt2*, *plap3*, and *plap6* were highest at 50 DAF, whereas *tpt, ppt1*, and *plap4* were highest at 70 DAF (Fig. 6). Six unigenes were found for ACBP (three for ACBP4 and two for ACBP5), whereas seven were found for ABC (one for ABCA9, five for ABCD1, and one for ABCI13). The expression of *acbp4* had a peak at 70 DAF, while *acbp5* peaked at 100DAF. The three *abc* unigenes showed an up-down-up change trend during kernel development (Fig. 6).

Unigenes identified to encode relevant transcription factors (TFs) included abscisic acid insensitive 3 (ABI3), abscisic acid insensitive 4 (ABI4), fusca3 (FUS3), glabra 2 (GL2), high-level expression of sugar-inducible gene 2 (HSI2/VAL1), HSI2-like1 (HSIL1/VAL2), HSI2-like2 (HSIL2/VAL3), leafy cotyledon 1(LEC1), LEC1-like (L1L), leafy cotyledon2 (LEC2), pickle (PKL), and wrinkled 1 (WRI1). Twenty-one unigenes were found for 11 of these TFs (belonging to six families), including ABI3 (two unigenes), ABI4 (one unigene), FUS3 (one unigene), GL2 (one unigene), HSI2/VAL1 (three unigenes), HSIL1/VAL2 (two unigenes), HSIL2/VAL3 (two unigenes), LEC1 (one unigene), L1L (one unigene), PKL (five unigenes), and WRI1 (two unigenes). However, no unigenes showed homology to LEC2. Among these TFs, unigenes for ABI3, ABI4, HSI2/VAL1, L1L, PKL and WRI1 had peak expression at 70 DAF, while unigenes for GL2 and LEC1 peaked at 50 DAF (Fig. 6). Unigenes for FUS3, HSIL1/VAL2 and HSIL1/VAL3 had low expression levels during the whole of kernel development.

Five enzymes related to carbon partitioning in the glycoxidation pathway were analyzed: glucose-1-phosphate adenylyltransferase (APG, EC: 2.7.7.27), starch synthase (SS, EC: 2.4.1.21), glucose-6-phosphate isomerase (GPI, EC: 5.3.1.9), glyceraldehyde 3-phosphate dehydrogenase (GAPDH, EC: 1.2.1.12), and malate dehydrogenase (MDH, EC:1.1.1.37). There were nine, seven, five, four and seven unigenes identified to encode APG, SS, GPI, GAPDH and MDH, respectively. All except APG had unigene expression peaks at 70 DAF (Fig. 6).

## Discussion

### Oil accumulation and biodiesel fuel property assessment in developing *S. tonkinensis* kernels

Oil content is a principal factor for evaluating the potential of biodiesel feedstocks [4, 9]. Previous studies have shown that the oil content of fully ripened *S. tonkinensis* kernels ranges from 58.6 to 63.0% [9, 14, 15], which is higher than many other oil plants including *L. glauca* (31.6%), *P. chinensis* (35.1%), *J. curcas* (39.8%), and *Vernicia fordii* (Hemsl.) Airy Shaw (40.0%) [32, 35–37]. In this study, the dynamic pattern of oil content based on dry mass showed an up-down-up trend with peaks at 80 and 140 DAF. Conversion of these data relative to fresh mass, based on water contents at corresponding times in Zhang et al. [15], resulted in a very similar pattern (*ρ* = 0.936, *p* < 0.01). A rapid increase in oil content from 60 to 70 DAF coincides with the initiation of rapid growth in kernel biomass. Thereafter, over the period where kernel biomass is known to increase steadily [15], oil content fluctuated (Fig. 1a). However, considering both content and size, maximum oil yield is expected by 140 DAF.

In addition to quantity, feedstock quality, as indicated by the proportion of unsaturated FAs, especially C18:1 and C18:2, is critically important [38, 39]. During kernel development, the major FA composition was steady and in very close agreement with previous analyses of *S. tonkinensis* kernel oil [15], with C18:1 and C18:2 together accounting for 86% of the total FA content. Given that oil content and composition are both relatively stable by 130 DAF, the best harvest time for *S. tonkinensis* kernels will correspond with maximum yield, which is reached at 140 DAF [15].

**Fig. 7:**
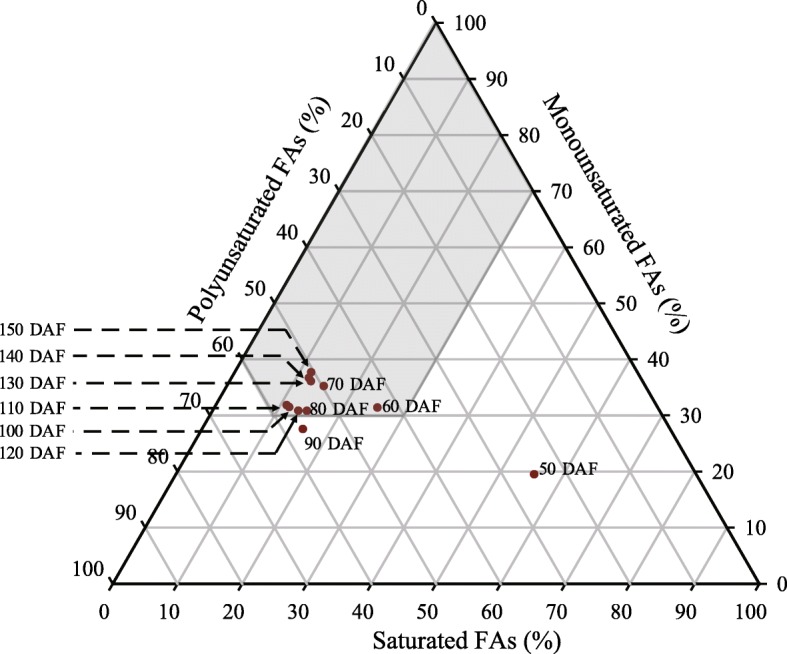
Placement of kernel oil samples on a chart predictive of biodiesel properties based on FA composition. The gray area delineates FA compositions satisfying limits for biodiesel fuel properties

Important biodiesel fuel properties including density (ρ), kinematic viscosity (η), cetane number (CN), iodine value (IV), and cold filter plugging point (CFPP) are critical indicators for the utilization of woody biodiesel species and can be predicted from the FA composition of the feedstock [9, 32, 40]. For *S. tonkinensis* in the present study, the estimated average ρ, η, CN, IV, and CFPP values at kernel maturity are 0.86 kg/m^3^, 3.40 mm^2^/s 48.2, 118.3 g/100 g and − 9.80 °C, respectively (Additional file 1). Indicators CN, IV and CFPP are considered satisfied when the percentages of saturated, monounsaturated, and polyunsaturated acids are within the shaded region of a triangular prediction model (Fig. 7) [40]. With the exceptions of 50 and 90 DAF, the kernel FA composition is always in this region. Wu et al. [9] have previously shown that *S. tonkinensis* kernel oil from mature seed satisfies the biodiesel standards of China (GB/T 20828), the European Union (EN 14214), Germany (DIN V51606), and the USA (ASTM D6751). In the present study, ρ, η and IV values of oils from mature *S. tonkinensis* kernels meet all four standards, while the CN and CFPP only meet standards ASTM D6751 and DIN V51606, respectively.

### Clustering analysis of DEGs

According to the functional annotation and classification of the unigenes and KEGG pathway assignment [31, 33], multiple unigenes encoding key enzymes associated with oil accumulation were identified. All unigenes that were differentially expressed between different sampling dates, irrespective of pathway assignment, were grouped into 9 clusters based on their temporal expression patterns (Additional file 3). These clusters show distinct patterns in expression throughout kernel development, but also significant temporal overlap in terms of associated pathways and processes. For example, clusters I and II represented DEGs with high expression at 50 DAF and were enriched in DEGs associated with amino acid biosynthesis, whereas DEGs related to FA biosynthesis were mainly found in clusters III and IV which represented the unigenes having high expression levels mostly at 70 DAF. These patterns are consistent with protein accumulation beginning sooner than FA accumulation [15].

The DEGs related to TAG biosynthesis were mainly found in clusters V, VII and IX. Clusters V and VII represented the unigenes having high expression levels that were delayed relative to clusters III and IV, but there was no clear temporal relationship with oil accumulation for cluster IX. Furthermore, the unigenes in cluster VIII, whose expression change trends best coincided with the up-down-up pattern of oil accumulation during kernel development, were obviously enriched in glycolysis/gluconeogenesis, starch and sucrose metabolism and protein process in endoplasmic reticulum rather than FA or TAG biosynthesis. It’s possible that some of these glycolysis/gluconeogenesis and starch and sucrose metabolism-related unigenes may be involved in the provision of substrate to support both phases of FA biosynthesis.

### Pyruvate and acetyl-CoA formation

To support FA biosynthesis, sucrose, the major form of sugar transported in most plants, is decomposed into fructose and glucose to yield pyruvate by way of the glycolytic pathway [19, 21, 41]. Three enzymes in this pathway, PFK, PFP, and PK, play key regulatory roles in the formation of pyruvate [21, 29]. Pyruvate is used to produce acetyl-CoA by the PDC directly, and ACS can also produce acetyl-CoA by utilizing acetate produced from pyruvate by way of acetaldehyde. In addition, the PPP operates parallel to glycolysis, starting at G6PD, while also supplying fatty acid biosynthesis with NADPH by way of PDG [19, 21].

The enzymes PFK and PFP both catalyze the phosphorylation of fructose-6-phosphate, the first committed step of glycolysis, but PFK has been identified in both cytosol and plastid, while PFP is restricted to plastids [28, 42]. In concurrence, G6PD1, which is cytosolic, had a unigene expression pattern similar to PFK, while G6PD2, which is located in the plastid, had a unigene expression pattern similar to PFP. These expression patterns are consistent with the existence of phosphohexose in both cytosol and plastids. The dynamic patterns of most unigenes encoding PK were coincident with *pfp*, including *pk1*, *pk2*, *pk4, pk6* and *pk10*, which are restricted to the plastid. The unigenes for plastidial PDC E1-α and PDC E2 had similar patterns, as did ACS, indicating that acetyl-CoA could be formed by two different pathways, which contrasts with results in *P. sibirica* that suggested a less important role for ACS [29]. Like *pfk*, and consistent with *P. sibirica*, expression patterns of unigenes for non-plastidial PDC E1-β and PDC E3 trended downwards from 50 DAF. Most unigenes related to pyruvate and acetyl-CoA formation in the glycolytic pathway had higher expression levels at the first two time points (50 DAF and 70 DAF), indicating greater emphasis on the formation of these primary substrates during the early stages of *S. tonkinensis* kernel development.

### Fatty acid biosynthesis and oil accumulation

**Fig. 8:**
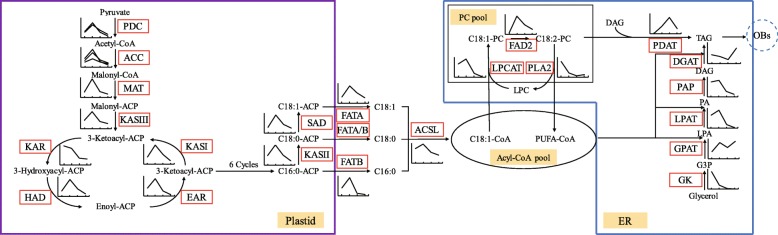
Overview of the pathway and temporal expressional patterns of oil accumulation. Icons accompanying enzymes show the expression trends of associated unigenes. Abbreviations: PDC, pyruvate dehydrogenase complex; ACC, acetyl-CoA carboxylase; MAT, malonyl-CoA-ACP transacylase; KASI, 3-oxoacyl-ACP synthase I; KASII, 3- ketoacyl-ACP synthase II; KASIII, 3-ketoacyl-ACP synthase III; KAR, 3-ketoacy-ACP reductase; HAD, 3-hydroxyacyl-ACP dehydratase; EAR, enoyl-ACP reductase I; SAD, stearoyl-ACP desaturase; FATA, acyl-ACP thioesterase A; FATB, acyl-ACP thioesterase B; ACSL, long-chain acyl-CoA synthetases; LPCAT, lysophosphatidylcholine acyltransferase; FAD2, fatty acid desaturase 2; PLA2, phospholipase A2; GK, glycerol kinase; GPAT, *sn*-G3P acyltransferase; LPAT, lysophosphatidyl acyltransferase; PAP, phosphatidic acid phosphohydrolases; DGAT, acyl-CoA: DAG acyltransferase; PDAT, phospholipid: diacylglycerol acyltransferase

Focusing on key enzymes involved in oil biosynthesis and accumulation, a summary overview of oil accumulation processes in developing kernels was constructed (Fig. 8). The de novo FA biosynthesis from precursors of acetyl-CoA occurs in plastids and adds two carbon units to malonyl-acyl carrier protein (ACP) in every condensation reaction cycle catalyzed by the FAS complex [43–45]. ACC, as the first committed key and a rate-limiting enzyme, converts acetyl-CoA to malonyl-CoA [29]. MAT converts malonyl-CoA to malonyl-ACP, which is the primary substrate for a subsequent series of condensation reactions [45]. In each condensation cycle, the FAS complex adds two additional carbon units to malonyl-ACP [46] to yield either palmitic acid-ACP (16:0-ACP) or stearic acid-ACP (18:0-ACP) after six or seven cycles, respectively. Additionally, SAD converts 18:0-ACP to oleic acid-ACP (18:1-ACP). Then, FAT releases free FA from ACP; specifically, FATA releases free C18:1 from C18:1-ACP, and FATB releases free C16:0 from C16:0-ACP, while either FATA or FATB can release C18:0 from C18:0-ACP. ACSL on the outer membrane of the plastid esterifies free FAs to generate an acyl-CoA pool [19, 47]. After that, some acyl-CoAs will combine with glycerol-3-phosphate (G3P) to form TAG, while others, especially C18:1-CoA, will enter the phosphatidylcholine (PC) pool and be esterified to form acyl-PCs by LPCAT. In the PC pool, FAD2 converts C18:1-PC to C18:2-PC, an additional substrate for TAG biosynthesis [31, 46]. PLA2 recycles PC to lysophosphatidylcholine (Lyso-PC) [46].

In this study, most FA biosynthesis unigenes had dynamic expression patterns similar to the unigenes related to pyruvate and acetyl-CoA formation; however, the rise in expression levels from 50 DAF to 70 DAF was in some cases more pronounced for FA biosynthesis-related enzymes (i.e., MAT, HAD, EAR, and SAD) than for unigenes related to pyruvate and acetyl-CoA formation, in accordance with the carbon flow from sugar to oil [15, 19]. The unigenes of FATB, the main enzyme responsible for free saturated FA release, had low expression levels at the last two time points (100 and 130 DAF), consistent with the actual FA profiles (especially C16:0 and C18:0) reported in Fig. 1b. The unigenes of FAD2, the main enzyme responsible for desaturation in unsaturated FA biosynthesis [48], showed a rapid rise in expression levels between 50 and 70 DAF and then a relatively slow decline later on, consistent with the actual FA profiles (cf., the increase in unsaturated relative to saturated FAs from 50 to 70 DAF in Fig. 1b, d). The identification of *StFAD2* would be useful in genetic engineering of *S. tonkinensis* for producing seed oils optimal for biodiesel fuel properties.

TAG is the major form of carbon storage in oil seeds. De novo TAG biosynthesis from precursors of G3P occurs in the ER via the Kennedy pathway [20, 49]. TAG is stored in oil bodies surrounded by a lipid monolayer and associated oil-body-membrane proteins including OLE, CLE and SLE, which are released from the ER into the cytoplasm [50, 51]. GPAT, as the first committed key enzyme in the Kennedy pathway, catalyzes G3P acylation to form lysophosphatidic acid (LAP) in the *sn*-1 position [52]. LPAT catalyzes LAP acylation to form phosphatidic acid (PA) in the *sn*-2 position. PAP converts PA to diacylglycerol (DAG). After that, rate-limiting enzymes DGAT and PDAT catalyze DAG acylation at the *sn*-3 position with acyl-CoA or acyl-PC to form TAG [47, 53]. In addition, G3P is converted from glycerol by GK [31].

Among the 13 *gpat* unigenes, the sustained expression of *gpat9* in *S. tonkinensis* kernels suggests that GPAT9 is the key initial acyltransferase for TAG biosynthesis [29, 53]. In contrast, *gpat8*, involved in cutin biosynthesis [54], was strongly expressed early in kernel development (50 and 70 DAF). The unigenes related to PC, PA and DAG biosynthesis such as *lpcat*, *fad2*, *pla*2, *gk*, *gpat9*, *lpat2*, and *pap* also had peaks at the earlier stages (50 DAF or 70 DAF), whereas the unigenes related to TAG biosynthesis such as *dgat* and *pdat2*, peaked later on (100 DAF or 130 DAF) (Figs. 6 and 8). The maximum expression of *pdat2* and *dgat* was at 100 DAF and 130 DAF (Fig. 8), respectively, indicating that TAG biosynthesis was accomplished both by DGAT with acyl-CoA via the Kennedy pathway and by PDAT with acyl-PC from the PC pool [31, 55].

As FA and TAG accumulated during kernel development, unigenes related to oleosin synthesis (*ole1*, *ole3* and *ole5*), and thus oil body formation, were steeply and steadily upregulated (Fig. 6). In particular, the expression level of *ole1* was very high. In contrast, *cle* expression remained quite low throughout kernel development. These patterns are consistent with results reported for developing seed kernels of *P. sibirica* [29]. The expression pattern of *ole1* was very similar to the evolution of total soluble protein content during kernel development reported by Zhang et al. [15].

### Fatty acid and triacylglycerol degradation and metabolism

During times of carbon and energy deficiency in higher plants, TAG is hydrolyzed to release free FAs and glycerol, which is vital for cellular energy balance, lipid homeostasis, membrane proliferation, cell growth, and survival [56]. There are two TAG lipases, TAGL and MAGL, responsible for the majority of oil breakdown [31, 56]. The released free FAs and/or acyl-CoA esters are converted to acetyl-CoA in the glyoxylate cycle and gluconeogenesis via the β-oxidation pathway with three core enzymes consisting of ACOX, MFP2 and ACAA [57]. In *S. tonkinensis*, the expression patterns of unigenes related to FA and TAG degradation and metabolism had peaks at 70 DAF (Fig. 6), similar to the major unigenes associated with FA and TAG biosynthesis. Consequently, the unigenes for DGAT, PDAT, and TAGL, three vital enzymes in the TAG pathway, do not have the same expression pattern, suggesting opportunities for genetic cloning and modification to improve cellular oil content and quality. ACOX1, active in the desaturation of both long- and medium-chain acyl-CoAs, and ACOX2, targeting long-chain acyl-CoAs, showed high unigene expression levels early in kernel development, indicating that free FA (especially C16:0 and C18:0) may be broken down to provide acetyl-CoA, the key metabolite for energy production.

### Transmembrane transporters and transcription factors involved in oil accumulation

In general, plant glycolysis occurs in both cytosol and plastid, while FA and TAG biosynthesis occur in plastid and ER, respectively [43, 45]. Therefore, the interchange of glycolytic intermediates and FA between different organelles becomes necessary. Several highly selective plastidic transporters, including GLT, GPT, TPT, PPT and PLAP, transport glycolytic metabolites generated in the cytosol to plastids for plastidial glycolysis [19, 21, 24, 29, 58]. ACBP and ABC are possible transporters related to FA or acyl-CoA transfer [23, 59]. In the present study, expression levels of *glt1* and *gpt2* were highest at 50 DAF, whereas *tpt* and *ppt1* were highest at 70 DAF, the latter showing a pattern similar to glycolysis-related unigenes in developing kernels (Figs. 1 and 6). These RPKM levels and patterns suggest that the main carbon source for FA biosynthesis in *S. tonkinensis* kernels is triosephosphate originating from phosphohexose outside the plastid, despite relatively high *pfp* expression at all points of sampling. Expression of PLAP6, which may be related to plastid formation [60], was largely restricted to earlier stages. Expression levels of unigenes for ACBP4 and ACBP5, as intracellular carriers of acyl-CoA esters [59], were both sustained through 70 to 100 DAF, suggesting they transport acyl-CoA to the ER during oil accumulation. The unigene for ABCA9, which also transports FA or acyl-CoA into the ER [23], had a small peak at 70 DAF but greater expression at the last time point, verifying that ABCA9, as well as ACBP4 and ACBP5, plays an important role when storage lipids accumulate in developing kernels.

According to previous studies, TFs play positive or negative roles in seed oil synthesis and deposition [61–65]. In this study, expression patterns for ABI3, ABI4, HSI2/VAL1, L1L, PKL, and WRI1 suggest that they play positive roles in regulating genes for oil biosynthesis, while other TFs may act as negative or ambiguous regulatory factors. Among them, WRI1 displayed a coordinated transcriptional profile with *pk*, *pdc*, *acc*, *kas*, *sad*, and *fad2*, consistent with its function as a central regulator that directs carbon flux from glycolysis into FA biosynthesis during seed oil accumulation [65, 66]. Similar results were seen in previous investigations on *E. guineensis* and *P. sibirica* [28, 29].

### Carbon competition during kernel development

In developing seeds, carbon partitioning determines the eventual starch/oil balance [44]. Zhang et al. [15] suggested that there is carbon competition between fatty acid and starch biosynthesis during *S. tonkinensis* kernel development. They found that between 40 and 60 DAF, the total soluble sugar concentration increased from 120 to 200 mg/g FW, but it then decreased to approximately the initial level by 90 DAF, where it remained until kernel maturation. Concurrently, starch concentration remained low (about 3 mg/g FW) between 30 and 80 DAF but then began to increase steadily to 18 mg/g FW. This phenomenon may result from the combined action of a series of enzymes related to glycoxidation, such as APG, SS, GPI, GAPDH, MDH and the PDC. The expression patterns of the unigenes for these enzymes were similar to changes in their respective activities reported in earlier research [15]. The unigenes for SS, the PDC and ACC, which are associated with the biosynthesis of starch or fatty acid, had peaks at 70 DAF. However, the subsequent decrease in expression levels of *pdc* and *acc* at 100 and 130 DAF were more obvious than for *ss*, and this might favor greater carbon flux to starch accumulation at middle to later stages of kernel development.

## Conclusions

In this study, the accumulation of oil content during kernel development showed an up-down-up pattern with two peaks at 80 and 140 DAF, respectively, while FA composition remained relatively static. Kernel oil from mature seed had good biodiesel fuel properties. Consequently, both oil quantity and quality are maximized if kernels are harvested when development is complete. Four time points were selected for further transcriptome analysis, and a total of 168,205 unigenes were assembled and annotated successfully. Differentially expressed unigenes were grouped into 9 clusters based on their expression patterns. Three main patterns of expression in unigenes related to oil accumulation were evident. The majority of unigenes encoding enzymes associated with FA biosynthesis peaked at 70 DAF, on the cusp of a rapid increase in oil content. Expression of these unigenes then declined to lower levels while oil synthesis was sustained during kernel growth. Unigenes peaking or with relatively high expression at 50 DAF tended to be associated with plastid formation and earlier stages of oil synthesis in the cytosol, such as pyruvate and acetyl-CoA formation (directly from pyruvate and indirectly via acetaldehyde), indicating that the main carbon source for FA biosynthesis is triosephosphate originating from phosphohexose outside the plastid. Relatively few unigenes peaked at 100 or 130 DAF, but notable exceptions were unigenes associated with TAG biosynthesis and oil body development. TAG biosynthesis is likely accomplished both by DGAT via the Kennedy pathway and by PDAT from the PC pool. Expression patterns were also consistent with some carbon competition for starch accumulation at middle to late stages of kernel development, as reported by Zhang et al. [15].

This is the first comprehensive analysis of transcriptome and sequence information for genes related to oil accumulation in developing *S. tonkinensis* kernels. Results of this study will serve as an important foundation to more deeply explore the regulatory mechanism of oil accumulation in this species, and may provide reference for other potential biodiesel species.

## Methods

### Plant materials and RNA extraction

The *S. tonkinensis* plants (from Jishui, Jiangxi Province) used in this study were purchased by Jiangsu Guoxing Co., Ltd., in 2011, and planted in the *Styracaceae* Germplasm Repository, Luhe District, Nanjing, China (32°32′ N, 118°50′ E), where they grew under natural conditions. In early May, 2017, 15 trees were tagged for sampling. Beginning July 15th (50 DAF), fresh fruits were randomly collected every 10 days. The kernels stripped from fruits and seed coats were immediately frozen in liquid nitrogen and stored at − 70 °C until use. After drying in a 65 °C oven for 72 h and weighing, oil content and FA composition, including proportions of saturated, monounsaturated and polyunsaturated FA, were determined by extraction on a Soxhlet apparatus followed by gas chromatography-mass spectrometry (GC/MS), using methods previously described by Zhang et al. [15]. Biodiesel fuel properties including density (ρ), kinematic viscosity (η), cetane number (CN), iodine value (IV), and cold filter plugging point (CFPP) were predicted from the FA composition according to Wang et al. [40] and expressed using a triangular prediction model based on the percentages of saturated, monounsaturated, and polyunsaturated acids [40].

Kernels from four representative time points were used for comparative deep transcriptome analysis. There were three biological replicates for each time point, with each replicate consisting of material pooled from five different trees. Total RNA was extracted using Plant RNA Kit (Omega Bio-Tek, Doraville, GA, USA) according to the manufacturer’s instructions. The quantity and quality of total RNA were assessed using 1% agarose gels and a Nanodrop ND 2000 Spectrophotometer (Nanodrop Technologies, Wilmington, DE, USA). The integrity and concentration of total RNA were assessed using a Bioanalyzer 2100 RNA 6000 Nano Kit (Agilent Technologies, Santa Clara, CA, USA).

### cDNA library construction and sequencing

The poly(A) mRNA was isolated from total RNA using an Oligotex mRNA Mini Kit (Qiagen, Inc., Valencia, CA, USA) according to the manufacturer’s instructions. cDNA library construction and normalization were performed using methods previously described by Niu et al. [29]. The 12 cDNA libraries were sequenced on the Illumina Hiseq 4000 Sequencing platform (Illumina, Inc., San Diego, CA, USA) and 150 bp paired-end reads were generated. The raw data were processed by removing the low-quality sequences (reads with more than 50% Q < 19 bases), the adaptor-pollute sequences, and sequences with ambiguous base reads accounting for more than 5%. The clean reads were assembled into unigenes by Trinity software (Trinity Release v2.4.0, Broad Institute of MIT and Harvard, Cambridge, MA, USA) [67].

### Functional annotation of unigenes

To understand their functions, the assembled unigenes were aligned to the publicly available protein databases including the NCBI non-redundant protein (NR), the Swiss-Prot protein (SwissProt), Gene Ontology (GO), Clusters of Orthologous Groups (COG), and the Kyoto Encyclopedia of Genes and Genomes (KEGG) using Basic Local Alignment Search Tool (BLAST) with a cut-off E-value of 10^− 5^ [68].

### Expression analysis of unigenes

The expression levels of the unigenes were calculated as Reads Per Kilobase Million Mapped Reads (RPKM), which eliminates the effect of sequencing depth and gene length on gene expression levels and permits direct data comparisons by the DESeq method [69]. Expression levels of unigenes involved in metabolic pathways associated with seed oil accumulation were calculated. The DEGs between different time points were identified with *p*_adj_ < 0.05 and |log2 (foldchange value) | ≥ 1 [69]. Clustering analysis using Mfuzz [70] was performed based on the expression patterns of all the identified DEGs (https://www.omicsolution.org/).

### qRT-PCR validation

Twelve unigenes related to oil accumulation were selected for validation via quantitative real-time PCR (qRT-PCR). The amplification primers were designed by Primer Premier 5.0 software (Premier Biosoft International, Palo Alto, CA, USA). All reactions were carried out on a StepOne Real-Time PCR System (Applied Biosystems, Foster City, CA, USA) using SYBR Green Dye (Takara, Dalian, China) according to the manufacturer’s instructions. Relative gene expression was calculated by the 2^-ΔΔCt^ method with 18S ribosomal RNA as an internal control [32]. All primers used in this study are listed in Additional file 6.

## Supplementary information

**Additional file 1. **Predicted biodiesel fuel properties of developing *S. tonkinensis* kernels.

**Additional file 2.** List of differentially expressed unigenes (DEGs) between different time points.

**Additional file 3.** Clustering analysis of all DEGs using Mfuzz. The red, green and blue colors indicate the match degrees between changes of genes and the major changes of the clusters. Red, green and blue represent high, moderate and low match degrees respectively.

**Additional file 4. **Validation of temporal unigene expression patterns uncovered by RNA-Seq. Panels show relative expression levels determined by qRT-PCR and RPKM values by RNA-Seq (50 DAF as the control) of 12 key genes (*accC*, *mat*, *had*, *kasI*, *kasII*, *sad*, *fatA*, *fatB*, *acp*, *gpat9*, *dgat*, *pdat2*). These results confirm that *accC*, *mat*, *had*, *kas*, *sad*, and *fat* for FA biosynthesis, and *dgat* and *pdat2* for TAG biosynthesis, had high expression at about 70DAF and 100 to 130 DAF, respectively.

**Additional file 5.** Annotation and expression of key unigenes related to oil accumulation.

**Additional file 6.** The qRT-PCR primer sequences.

## Data Availability

The Sequence data are deposited at the in the NCBI Sequence Read Archive (SRA) under accession numbers SRR8832448, SRR8832449, SRR8832450, SRR8832451, SRR8832452, SRR8832453, SRR8832454, SRR8832455, SRR8832456, SRR8832457, SRR8832458 and SRR8832459 (https://www.ncbi.nlm.nih.gov/bioproject/PRJNA530204). All data analyzed during this study are included in this published article and its supplementary information files.

## Abbreviations

ABC: ATP-binding cassette transporters
ABI3: Abscisic acid insensitive 3
ABI4: Abscisic acid insensitive 4
ACAA: Acetyl-CoA acyltransferase
ACBP: Acyl-CoA binding protein
ACC: Acetyl-CoA carboxylase
ACOX: Acyl-CoA oxidase
ACP: Acyl carrier protein
ACS: Acetyl-CoA synthetase
ACSL: Long-chain acyl-CoA synthetases
AGP: Glucose-1-phosphate adenylyltransferase
BLAST: Basic Local Alignment Search Tool
C16:0: Palmitic acid
C18:0: Stearic acid
C18:1: Oleic acid
C18:2: Linoleic acid
CLE: Caleosin
COG: Clusters of Orthologous Groups
DAF: Days after flowering
DAG: Diacylglycerol
DGAT: Acyl-CoA:DAG acyltransferase
EAR: Enoyl-ACP reductase
ER: Endoplasmic reticulum
FA: Fatty acid
FAD2: Fatty acid desaturase2
FAS: Fatty acid synthase
FAT: Acyl-ACP thioesterase
FUS3: Fusca3
G3G: Glycerol-3-phosphate
G6PD: Glucose-6-phosphate dehydrogenase
GAPDH: Glyceraldehyde 3-phosphate dehydrogenase
GC: Guanine-cytosine
GC/MS: Gas chromatography-mass spectrometry
GK: Glycerol kinase
GL2: Glabra 2
GLT: Glycolipid transporters
GO: Gene Ontology
GPAT: *sn*-G3P acyltransferase
GPI: Glucose-6-phosphate isomerase
GPT: Glucose-6-phosphate transporters
HAD: 3-hydroxyacyl-ACP dehydratase
HSI2/VAL1: High-level expression of sugar-inducible gene 2
HSIL1/VAL2: HSI2-like1
HSIL2/VAL3: HSI2-like2
KAR: 3-ketoacy-ACP reductase
KASI: 3-oxoacyl-ACP synthase I
KASII: 3-ketoacyl-ACP synthase II
KASIII: 3-ketoacyl-ACP synthase III
KEGG: Kyoto Encyclopedia of Genes and Genomes
L1L: LEC1-like
LAP: Lysophosphatidic acid
LEC1: Leafy cotyledon 1
LPAT: Lysophosphatidyl acyltransferase
LPCAT: Lysophosphatidylcholine acyltransferase
MAGL: Monoacylglycerol lipase
MAT: Malonyl-CoA-ACP transacylase
MDH: Malate dehydrogenase
MF2: Enoyl-CoA hydratase/3-hydroxyacyl-CoA dehydrogenase
NCBI: National Center for Biotechnology Information
NGS: Next-generation sequencing
NR: Non-redundant protein
OLE: Oleosin
PA: Phosphatidic acid
PAP: Phosphatidic acid phosphohydrolase
PC: Phosphatidylcholine
PDAT: Phospholipid:diacylglycerol acyltransferase
PDC: Pyruvate dehydrogenase complex
PFK: ATP-dependent phosphofructokinase
PFP: Pyrophosphate-dependent phosphofructokinase
PGD: 6-phosphogluconate dehydrogenase
PK: Pyruvate kinase
PKL: Pickle
PLA2: Phospholipase A2
PLAP: Plastid-lipid-associated protein
PPT: Phosphoenolpyruvate transporters
qRT-PCR: Quantitative real-time PCR
RPKM: Reads per kilobase million mapped reads
SLE: Steroleosin
SRA: Short Read Archive
SS: Starch synthase
TAG: Triacylglycerol
TAGL: Triacylglycerol lipase
TF: Transcription factor
TPT: Triose phosphate transporters
WRI1: Wrinkled 1
