# Supplementary figures and images for: Transcriptome analysis of metabolic pathways associated with oil accumulation in developing seed kernels of Styrax tonkinensis, a woody biodiesel species
Source: BMC Plant Biol. 2020 Mar 18;20:121. doi: 10.1186/s12870-020-2327-4 (PMC7079523; doi:10.1186/s12870-020-2327-4)

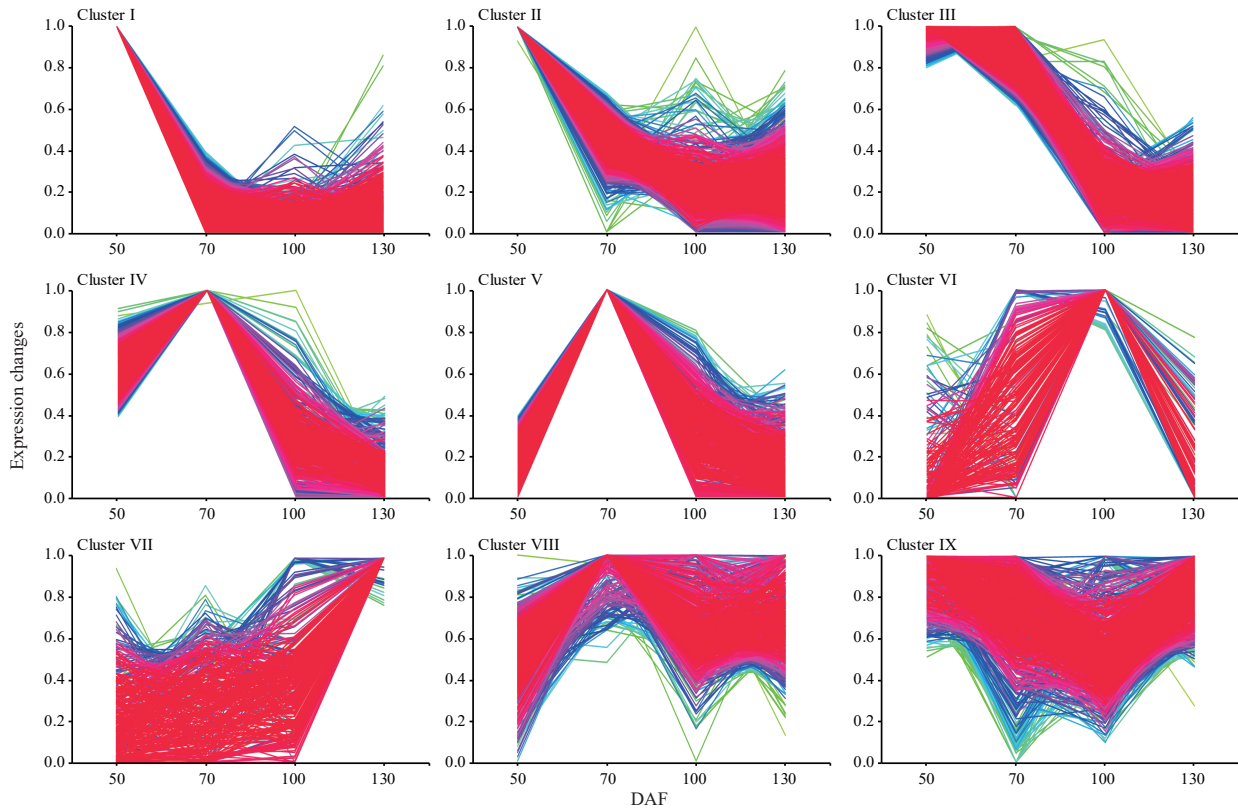

Supplement: Supplementary file 3 — Additional file 3. Clustering analysis of all DEGs using Mfuzz. The red, green and blue colors indicate the match degrees between changes of genes and the major changes of the clusters. Red, green and blue represent high, moderate and low match degrees respectively. [file 12870_2020_2327_MOESM3_ESM.pdf]

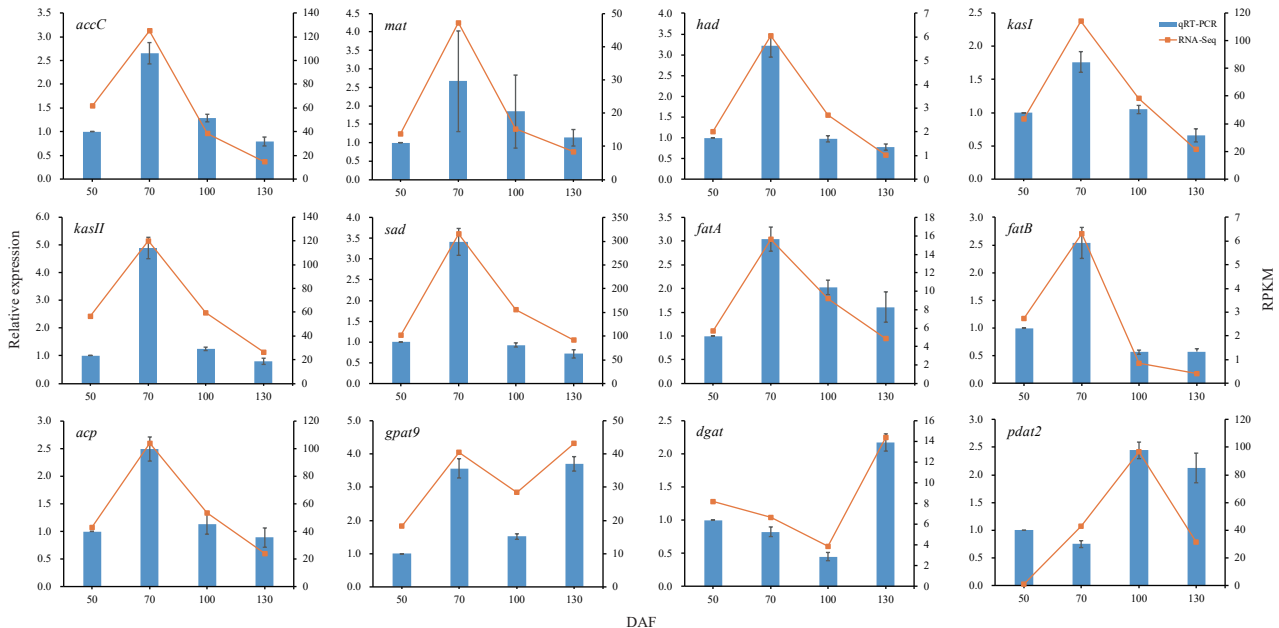

Supplement: Supplementary file 4 — Additional file 4. Validation of temporal unigene expression patterns uncovered by RNA-Seq. Panels show relative expression levels determined by qRT-PCR and RPKM values by RNA-Seq (50 DAF as the control) of 12 key genes (accC, mat, had, kasI, kasII, sad, fatA, fatB, acp, gpat9, dgat, pdat2). These results confirm that accC, mat, had, kas, sad, and fat for FA biosynthesis, and dgat and pdat2 for TAG biosynthesis, had high expression at about 70DAF and 100 to 130 DAF, respectively. [file 12870_2020_2327_MOESM4_ESM.pdf]
